# Supplementary material for: Evolutionary and ecological processes influencing chemical defense variation in an aposematic and mimetic Heliconius butterfly
Source: PeerJ. 2021 Jun 18;9:e11523. doi: 10.7717/peerj.11523 (PMC8216171; doi:10.7717/peerj.11523)
Supplement: Supplemental Information 1 [file peerj-09-11523-s001.pdf]

TITLE. Evolutionary and ecological processes influencing chemical defense variation in an aposematic and mimetic *Heliconius* butterfly

Supplementary File 1

Table S1A. Cyanogenic glucoside compound concentrations (mean, standard deviation (SD) and *P*-values of Tukey HSD pairwise comparisons) in natural populations of *Heliconius erato* and *Passiflora biflora* sampled along environmental gradients in Panama and Ecuador. Statistically significant pairwise comparisons are marked in bold.

	H. erato	Linamarin %							Lotaustralin %							Total cyanogen %						
				Pairwise comparisons (Tukey HSD *P*-value)							Pairwise comparisons (Tukey HSD *P*-value)							Pairwise comparisons (Tukey HSD *P*-value)				
**Country**	**Population**	Mean	SD	*Dry*	*Int*	*Wet*	*Low*	*High*	Mean	SD	*Dry*	*Int*	*Wet*	*Low*	*High*	Mean	SD	*Dry*	*Int*	*Wet*	*Low*	*High*
Panama	*Dry*	0.771	0.557						0.293	0.177						1.064	0.709					
Panama	*Intermediate*	0.776	0.465	0.997					0.495	0.218	**<0.001**					1.271	0.631	0.432				
Panama	*Wet*	0.569	0.356	0.445	0.295				0.337	0.198	0.766	**0.007**				0.907	0.521	0.905	0.098			
Ecuador	*Low*	0.011	0.028	**<0.001**	**<0.001**	**<0.001**			0.000	0.000	**<0.001**	**<0.001**	**<0.001**			0.011	0.028	**<0.001**	**<0.001**	**<0.001**		
Ecuador	*High*	0.026	0.033	**<0.001**	**<0.001**	**<0.001**	0.911		0.000	0.000	**<0.001**	**<0.001**	**<0.001**	1.000		0.026	0.033	**<0.001**	**<0.001**	**<0.001**	0.936	

	P. biflora	Passibiflorin (µg/mg)					Passibiflorin triglycoside (µg/mg)					Tetraphyllin A (µg/mg)					Total cyanogens (µg/mg)				
				Pairwise comparisons (Tukey HSD *P*-value)					Pairwise comparisons (Tukey HSD *P*-value)					Pairwise comparisons (Tukey HSD *P*-value)					Pairwise comparisons (Tukey HSD *P*-value)		
**Country**	**Population**	Mean	SD	*Dry*	*Int*	*Wet*	Mean	SD	*Dry*	*Int*	*Wet*	Mean	SD	*Dry*	*Int*	*Wet*	Mean	SD	*Dry*	*Int*	*Wet*
Panama	*Dry*	11.005	9.459				0.732	0.620				0.113	0.416				11.850	10.073			
Panama	*Intermediate*	20.291	12.925	**0.035**			1.166	0.654	0.126			0.087	0.289	0.970			21.544	13.586	**0.038**		
Panama	*Wet*	17.095	7.528	0.123	0.653		0.911	0.497	0.598	0.478		0.000	0.000	0.448	0.709		18.005	7.953	0.146	0.627	

Table S1B. Variance components of *Heliconius* biosynthesized cyanogenic toxicity estimated with REML animal models. For each variance component, Variance (V), Standard error (*S.E.*) of variance, Statistical significance (*P*) of variance, and Proportion of variance of total phenotypic variance (V/V_P) are shown. In addition, broad-sense heritability H^2 (V_G/V_P) and evolvability e_μ (V_G/μ^2; %) are calculated based on the genetic variance component (shaded columns). Data consists of 322 observations, including 20 mothers, 13 fathers, and 289 F1 offspring belonging to 20 broods (of which 18 are full-siblings, and 2 are paternal half-siblings).

			Genetic variance component, heritability and evolvability					Maternal variance				Fixed effects								Residual variance			Total phenotypic variance
												Feeding treatment				Sex							
Model type	Cyanogen trait	Trait mean (μ)	V_G	V_G *S.E.*	*P* V_G	H^2 (V_G/V_P)	e_μ (V_G/μ^2); %	V_{mat}	*S.E.* V_{mat}	P V_{mat}	V_{mat}/V_P	V_{trm}	*S.E.* V_{trm}	P_{trm}	V_{trm} / V_P	V_{sex}	*S.E.* V_{sex}	P_{sex}	V_{sex}/V_P	V_R	*S.E.* V_R	V_R/V_P	V_P
1	Total CNglc %	0.772	0.0093	0.0069	0.0368	0.1152	1.5548					0.001	7.E-05	0.467	0.011	0.001	7.E-05	0.057	0.011	0.070	0.008	0.874	0.0805
1	Linamarin %	0.577	0.0063	0.0043	0.0202	0.1387	1.8869					5.E-05	4.E-06	0.418	0.001	0.001	5.E-05	0.036	0.013	0.038	0.004	0.848	0.0453
1	Lotaustralin %	0.195	4.0E-04	3.9E-04	0.1304	0.0668	1.0394					5.E-05	4.E-06	0.621	0.008	3.E-05	2.E-06	0.224	0.004	0.005	0.001	0.929	0.0059
2	Total CNglc %	0.772	1.0E-07	0.0312	1.0000	1.4E-06	1.7E-05	0.007	0.013	1.000	0.102	0.003	2.E-04	0.134	0.043	2.E-04	1.E-05	0.378	0.002	0.065	0.018	0.895	0.0727
