# Supplementary material for: Evolutionary and ecological processes influencing chemical defense variation in an aposematic and mimetic Heliconius butterfly
Source: PeerJ. 2021 Jun 18;9:e11523. doi: 10.7717/peerj.11523 (PMC8216171; doi:10.7717/peerj.11523)
Supplement: Supplemental Information 2 [file peerj-09-11523-s002.pdf]

TITLE. Evolutionary and ecological processes influencing chemical defense variation in an aposematic and mimetic *Heliconius* butterfly

Supplementary File 2

Table S2: Summary of *Heliconius erato* broods.

	Mother id	Father id	Offspring n	Feeding treatment
	1031	N.A.	7	NO
	1049	1029	6	NO
	1059	1037	7	NO
	1062	N.A.	12	NO
	1070	N.A.	10	NO
	1076	1030	10	NO
	1083	1030	9	NO
	1126	N.A.	9	NO
	1129	1100	8	NO
	1142	N.A.	7	NO
	1685	N.A.	12	NO
	1687	1421	8	NO
	1691	1657	6	NO
	1799	1664	5	NO
	1346	1291	38	YES
	1404	1238	42	YES
	1446	1224	20	YES
	1850	1722	25	YES
	1860	1729	16	YES
	2193	2120	32	YES
**n**	**20**	**13**	**289**	
		**Total n:**	**322**	
